# Psychosocial and functional difficulties in older adults with chronic non-specific low back pain

**DOI:** 10.1186/s12877-026-07420-y

**Published:** 2026-04-13

**Authors:** Kevser Gursan

**Affiliations:** Faculty of Health Sciences, Department of Physiotherapy and Rehabilitation, Usak University, Uşak, Turkey

**Keywords:** Low back pain, Older adults, Participation, Functional status, Kinesiophobia, Depression

## Abstract

**Background:**

Chronic non-specific low back pain (CNLBP) is an important health problem affecting people of all ages in societies. This health problem increases especially with age and participation restrictions and quality of life are negatively affected accordingly. This study was conducted to investigate the participation levels, pain, kinesiophobia, functional status and depression among older adults with CNLBP.

**Method:**

This descriptive and correlational study included 115 cognitively healthy older people over the age of 65 who applied to a state hospital with CNLBP. Data were collected using the Demographic Information Form, Montreal Cognitive Assessment, Visual Analog Scale, Tampa Scale of Kinesiophobia, Back Pain Function Scale, Keele Participation Assessment Scale and Geriatric Depression Scale. Data were evaluated using descriptive statistics, independent sample t-test, correlation and regression analyses.

**Results:**

The mean age of the older adults participating in the study was 71.90 ± 6.64. According to the results obtained, it was found that as the level of social participation of the older adults decreased, their depression, kinesiophobia and pain levels increased and their functional levels decreased. It was determined that social participation could explain 45% of functional losses and 20–25% of psychological symptoms.

**Conclusion:**

According to the findings, CNLBP has critical effects not only on the physical but also on the psychosocial status of the older people. Therefore, incorporating psychosocial factors affecting older adults into holistic rehabilitation plans is key to the successful treatment of CNLBP.

**Trial registration:**

Not applicable.

**Supplementary Information:**

The online version contains supplementary material available at 10.1186/s12877-026-07420-y.

## Introduction

Low back pain (LBP) is one of the most common musculoskeletal problems worldwide, causing a decrease in quality of life and mobility limitations, especially in older individuals [1]. It is estimated that approximately 619 million people worldwide experienced LBP in 2020, and this number is expected to reach 843 million by 2050 due to population growth and aging [2]. Older adults are the population most affected by LBP, and LBP is a significant cause of morbidity in this age group [3]. As a result of the prevalence of LBP among the older adults, LBP is shown to be one of the most common reasons for hospitalization in older individuals [4]. Approximately 80–90% of older adults presenting to the hospital with complaints of LBP are diagnosed with non-specific low back pain (NLBP) [4, 5]. There is no known pathoanatomical cause for NLBP. However, it usually develops due to long-term and untreatable mechanical disorders in the lumbar region [5, 6]. These mechanical disorders include lumbosacral muscle strain, disc herniation, lumbar spondylosis, spondylolisthesis, spondylolysis, vertebral compression fractures, acute/chronic traumatic injuries and/or degeneration of peripheral soft tissues [7].

If left untreated for a long time, LBP can turn into chronic non-specific low back pain (CNLBP). CNLBP can lead to not only physical but also serious psychosocial consequences such as limited movement in daily life, decreased social participation, impaired functional performance, kinesiophobia and depression [8]. CNLBP can cause social isolation by limiting the participation of the older people in daily life activities [9]. In addition, fear of movement may also occur in older adults due to pain. These fears are that the pain will be constant, may develop due to activity and that movement will harm the body [10]. This attitude is defined as “avoidance behavior” and in this case, it can cause individuals to avoid daily activities and increase functional disabilities over time [8, 11].

Physical discomfort, functional limitations, low physical activity levels and reduced performance capacities can be seen in older adults with CNLBP [12]. This condition seriously affects the independence of older people with CNLBP, leading to a decrease in quality of life and a high degree of disability. The World Health Organization has stated that this disability limits participation in ways such as decreasing social relationships, staying at home more and decreasing recreational activities [13]. In addition, depending on the type and severity of pain, the mental state of older adults can be significantly affected and the risk of depression can increase. Depression limits the participation of older people in the clinical rehabilitation process and delays the response to treatment, making LBP chronic [14]. Therefore, depression is not only a psychological condition but also a critical condition affecting the functionality and performance of older people. Therefore, it is important to consider lumbar pain together with functionality and depression when evaluating them and to implement early screening/interventions.

Although CNLBP is an important global health problem, its physical and psychosocial effects on the older population in Turkey, the effects of pain in the chronic period, and its relationships with other factors are not sufficiently known. Depression, which is one of the psychological health problems of the older people in particular, can negatively affect the functional status of the lumbar region due to reasons such as avoidance of movement, decrease in muscle strength, lack of motivation, social restrictions, and decrease in daily life activities. The relationships between participation, pain, kinesiophobia, depression, and functional status in individuals with CNLBP need to be investigated multidimensionally. Knowing whether these variables are related to each other or how they affect each other will contribute to the organization of clinical practices and the efficient provision of health services. Thus, the treatment and rehabilitation processes applied to older individuals with CNLBP can be made more successful. This study aims to examine the relationship between the level of social participation, pain, kinesiophobia, depression, and functional status in older adults with CNLBP.

## Methods

### Study design

In line with the purpose determined for the study, descriptive and correlational types of quantitative research methods were designed. This study was reported according to the Strengthening The Reporting of Observational Studies in Epidemiology guide [15].

### Study place and time

The study was conducted in a state hospital in the Central Anatolia region of Turkey between December 27, 2019 and February 25, 2020.

### Study setting and sample

The universe of the study consisted of older people who applied to a hospital with complaints of CNLBP. The study sample consisted of 115 older individuals who applied with complaints of CNLBP. Inclusion criteria were as follows: being 65 years of age or older, having complaints of non-specific low back pain (NLBP) for at least three months, being literate, being a volunteer, and receiving at least 21 points from the Montreal Cognitive Assessment (MoCA). The exclusion criteria were as follows: having a history of back surgery, using regular painkillers, having hearing and vision impairment, and having undergone physical therapy and rehabilitation within the last month. A two-way hypothesis was established for the study and A priori power analysis was performed with G*Power 3.1. The effect size was determined as f²=0.10, 5% margin of error, and a minimum of 91 participants with a target of 80% statistical power. Considering the missing data, the study was completed with 115 participants by taking 25% more. According to the post hoc power analysis performed with 115 participants, it was calculated as 87.4%. This showed that the study had sufficient statistical power at the assumed effect size.

### Data collection tools

The patients participating in the study were administered the Demographic Information Form (DIF), MoCA, Visual Analog Scale (VAS) (both at rest and during activity), Tampa Scale of Kinesiophobia (TSK), Back Pain Function Scale (BPFS), Keele Participation Assessment (KPA) and Geriatric Depression Scale (GDS).

DIF includes information such as age, gender, height, weight, marital status, occupation, education level, exercise habits, disease history, surgical operation history and medication use [6, 12, 14, 16].

MoCA evaluates various cognitive dimensions such as attention, concentration, executive functions, memory, language, visual-spatial skills, abstract thinking, calculation and orientation. This scale was developed by Nasreddine et al. in 2005 and its Turkish validity and reliability were performed in 2009 [17, 18]. The lowest score that can be obtained from MoCA is 0, and the highest score is 30. Scores of 21 and above from the scale represent mentally healthy individuals. In the Turkish validity and reliability study of the scale, Cronbach’s alpha was reported as 0.83, and the test-retest reliability was 0.89.

VAS is a valid and reliable scale widely used in clinics to assess pain intensity. It consists of a 10 cm horizontal line. The 0 point on the line means “I have no pain”, and the 10 point means “I have unbearable pain” [19]. VAS scores were assessed both at rest and during activity.

BPFS is a self-report scale that evaluates the difficulties experienced by individuals in daily life activities due to LBP. BPFS evaluates the functionality level of individuals in activities such as turning in bed, getting up from a chair, walking, standing, bending, going up and down stairs, lifting objects, being able to work while standing, being able to work while sitting, reaching, housework and personal care. BPFS was developed by Stratford et al. in 2000 and its Turkish validity was made by Maras et al. in 2019 [20, 21]. The lowest score is 0 and the highest score is 60. Each item is scored between 0 (I cannot do it at all) and 5 (I can do it without any difficulty). Higher scores indicate better functional capacity, while lower scores indicate serious functional limitation. In the Turkish validity and reliability study of the scale, Cronbach’s alpha was found to be 0.91 and the test-retest reliability was found to be 0.96.

KPA is a scale that evaluates the social participation of patients. The scores that can be obtained from KPA are between 0 and 11. A score of 0 indicates that the individual does not experience any participation limitation; scores between 1 and 11 indicate that the individual has participation limitation in at least one activity. KPA was developed by Wilkie et al. in 2005 and later in 2022, its Turkish validity and reliability were determined by Gursan et al. [22, 23] (Appendix A, B). In the Turkish validity and reliability study of the scale, Cronbach’s alpha was found to be 0.65 and the test-retest reliability was found to be 0.63.

TSK is a scale developed to measure fear of injury during movement. It includes parameters such as fear of injury/re-injury and avoidance behavior in work-related activities. The lowest score can be obtained is 17 and the highest is 68. High scores indicate high levels of kinesiophobia [24, 25]. TSK was developed by Vlaeyen et al. in 1995 and later in 2011, its Turkish validity and reliability were determined by Yılmaz et al. In the Turkish validity and reliability study of the scale, Cronbach’s alpha was found to be 0.74 and the test-retest reliability was found to be 0.80.

GDS is a 30-question scale used to assess depression in older individuals. The scores that can be obtained from the scale vary between 0 and 30. 0–9 indicates normal, 10–19 indicates mild depression, and 20–30 indicates severe depression. GDS was developed by Yesavage et al. in 1983 and its Turkish validity and reliability were later determined by Ertan et al. in 1997 [26, 27]. In the Turkish validity and reliability study of the scale, Cronbach’s alpha was found to be 0.91 and the test-retest reliability was found to be 0.84.

### Data collection process

The data of the study was applied face to face to patients in a hospital environment. The data collection process took an average of 30 min. The entire process of the study is as follows: Problem determination (September–October 2019), literature review (September 2019-January 2020), creation of the research question (December 2019), determination of research and analysis methods (November-December 2019), data collection and analysis (December 2019-February 2020).

### Data analysis

The data obtained in the study were analyzed with the Statistical Package for Social Sciences for Windows 25.0 program. Descriptive statistical methods (mean, standard deviation, minimum and maximum values) were used in the evaluation of the data. The conformity of the data to normal distribution was checked with normality tests and kurtosis and skewness values. In order for the tests and results to be reliable, the scales must be reliable. In this context, the reliability of the scales and sub-dimensions used in the study was examined with Cronbach’s alpha. Pearson correlation analyses were performed to examine the relationships between continuous data. Correlation coefficients were interpreted with r values ​​as 0.00–0.19: very weak, 0.20–0.39: weak, 0.40–0.59: moderate, 0.60–0.79: strong, 0.80–1.00: very strong. Simple and hierarchical regression analyses were performed. Regression analyses were performed to examine the predictive effect of participation level on pain, functional status, kinesiophobia and depression. A separate model was established for each dependent variable (VAS, BPFS, TSK, GDS). Model fit was evaluated with F test. β coefficients in the model showed the direction and magnitude of the relationship between the variables. The Durbin-Watson coefficient was used to test whether there was autocorrelation. This coefficient was between 1.5 and 2.5, indicating that there was no relationship between the errors. The significance level was accepted as *p* < 0.05 in all tests.

## Results

### Demographic and clinical characteristics of older adults with CNLBP

Of the 115 participants in the study, 55.7% were female, 61.7% were married, and 42.6% were retired. 65.2% of the participants had chronic diseases, 41.73% had undergone non-lumbar surgery. 58.26% had never undergone surgery. The rate of those who were on regular medication was 56.5%. 28.7% of the participants smoked, 14.8% consumed alcohol, and 11% exercised regularly. When pain areas were examined, 39.1% reported pain in the waist and both legs, while 22.6% stated that they experienced pain only in the waist area (Table 1). The mean age of the patients was 71.90 ± 6.64 years, the mean height was 165.17 ± 9.10 cm, and the mean weight was 78.27 ± 11.22 kg. The mean frequency of low back pain (within 1 year) was 2.49 ± 1.93; the mean duration of low back pain was 6.60 ± 4.79 years; and the mean duration of intense pain was 3.41 ± 2.36 months (Table 1).

**Table 1:** Demographic and clinical characteristics of the study participants

Characteristics		n	%	X±SD (min-max)
Gender	Woman	64	55.7	
	Male	51	44.3	
Marital status	Married	71	61.7	
	Single	44	38.3	
Profession	Housewife	42	36.5	
	Officer	4	3.5	
	Worker-farmer	8	7.0	
	Tradesmen	2	1.7	
	Retired	49	42.6	
	Not working	10	8.7	
Education status	Literate	39	33.9	
	Primary School	42	36.6	
	Middle School	15	13.0	
	High School	13	11.3	
	University	6	5.2	
Chronic Disease Status	There is	75	65.2	
	No	38	34.8	
Surgery	Other surgery(except lumbar)	48	41.73	
	No	67	58.26	
Continuous use of medication	There is	65	56.5	
	No	50	43.5	
Smoking	Yes	33	28.7	
	No	82	71.3	
Regular exercise	Yes	13	11.3	
	No.	102	88.7	
Alcohol	Yes	17	14.8	
	No.	98	85.2	
Pain localization	Waist	26	22.6	
	Waist and hips	16	13.9	
	Waist and right leg	11	9.6	
	Waist and left leg	17	14.8	
	Waist, right and left leg	45	39.1	
Age (years)				71.90 ±6.64 (65-92)
Length (cm)				165.17±9.10 (140-187)
Weight (kg)				78.27±11.22 (58-103)
Frequency of low back pain (1 year)				2.49±1.93 (1-15)
Low back pain (years)				6.60±4.79 (1-25)
Intense pain (months)				3.41±2.36 (1-12)

*n *Number of participants, *% *percentage, *X *mean, *SD *Standart Deviation, *min *minimum, *max *maximum

### Mean scores of older adults with CNLBP from scales

According to the descriptive statistics of the scales used by older adults with CNLBP, VAS resting mean is 3.23 ± 1.84, VAS activity mean is 4.77 ± 2.14, BPFS mean is 31.70 ± 9.64, KPA mean is 24.14 ± 5.29, TSK mean is 45.90 ± 5.17 and GDS mean is 12.20 ± 5.67 (Table 2).

**Table 2:** Descriptive statistics of scales

Paramaters	Min	Max	X ± SD
VAS resting	0.00	8.00	3.23 ± 1.84
VAS activity	1.00	10.00	4.77 ± 2.14
BPFS	10.00	53.00	31.70 ± 9.64
KPA	13.00	38.00	24.14 ± 5.29
TSK	30.00	61.00	45.90 ± 5.17
GDS	2.00	28.00	12.20 ± 5.67

*VAS *Visual Analog Scale, *TSK *Tampa Scale of Kinesiophobia, *BPFS *Back Pain Function Scale, *KPA *Keele Participation Assessment, *GDS *Geriatric Depression Scale, *X *mean, *SD *Standart Deviation, *min *minimum, *max *maximum

### Relationship between mean scores of older adults with CNLBP from scales

In the analysis, a statistically significant, negative and moderate relationship was found between KPA and BPFS (*p* < 0.01; *r*=-0.676). Positive and moderate relationship between KPA and TSK; Positive and moderate significant relationships were found between KPA and GDS; positive and moderate significant relationships were found between KPA and VAS score at rest; and positive and moderate significant relationships were found between KPA and VAS score during activity (Table 3).

**Table 3:** Relationships between scales

		KPA	BPFS	TSK	GDS	VAS rest	VAS activity
KPA	r	1					
	p						
BPFS	r	-0.676	1				
	p	0.000*					
TSK	r	0.517	-0.527	1			
	p	0.000*	0.000*				
GDS	r	0.480	-0.550	0.435	1		
	p	0.000*	0.000*	0.000*			
VAS at rest	r	0.445	-0.513	0.305	0.260	1	
	p	0.000*	0.000*	0.001*	0.005*		
VAS activity	r	0.502	-0.604	0.576	0.370	0.487	1
	p	0.000*	0.000*	0.000*	0.000*	0.000*	

*VAS *Visual Analog Scale, *TSK *Tampa Scale of Kinesiophobia, *BPFS *Back Pain Function Scale, *KPA *Keele Participation Assessment, *GDS *Geriatric Depression Scale, r: Pearson correlation coefficient, *p<0.05

A statistically significant, positive and moderate relationship was found between KPA and VAS score during activity (*p* < 0.01; *r* = 0.502). A statistically significant, negative and moderate relationship was found between BPFS and TSK (*p* < 0.01; *r*=-0.527). Similarly, there was a negative and moderate significant relationship between BPFS and GDS scores (*p* < 0.01; *r*=-0.550). Negative and moderate significant relationships were observed between BPFS score and VAS score at rest (*p* < 0.01; *r*= -0.513) and VAS score during activity (*p* < 0.01; *r*=-0.604) (Table 3).

A statistically significant, positive and moderate correlation was found between TSK and GDS (*p* < 0.01; *r* = 0.435). Positive and moderate correlations were also found between TSK and VAS resting (*p* < 0.01; *r* = 0.305) and VAS activity (*p* < 0.01; *r* = 0.576) scores. A weak but significant positive correlation was found between VAS resting score and GDS score (*p* < 0.01; *r* = 0.260). A moderate positive correlation was found between VAS resting and VAS activity scores (*p* < 0.01; *r* = 0.487), and a moderate positive correlation was found between VAS activity and GDS score (*p* < 0.01; *r* = 0.370) (Table 3).

### Predictors affecting participation of older adults with CNLBP

**Fig. 1:**
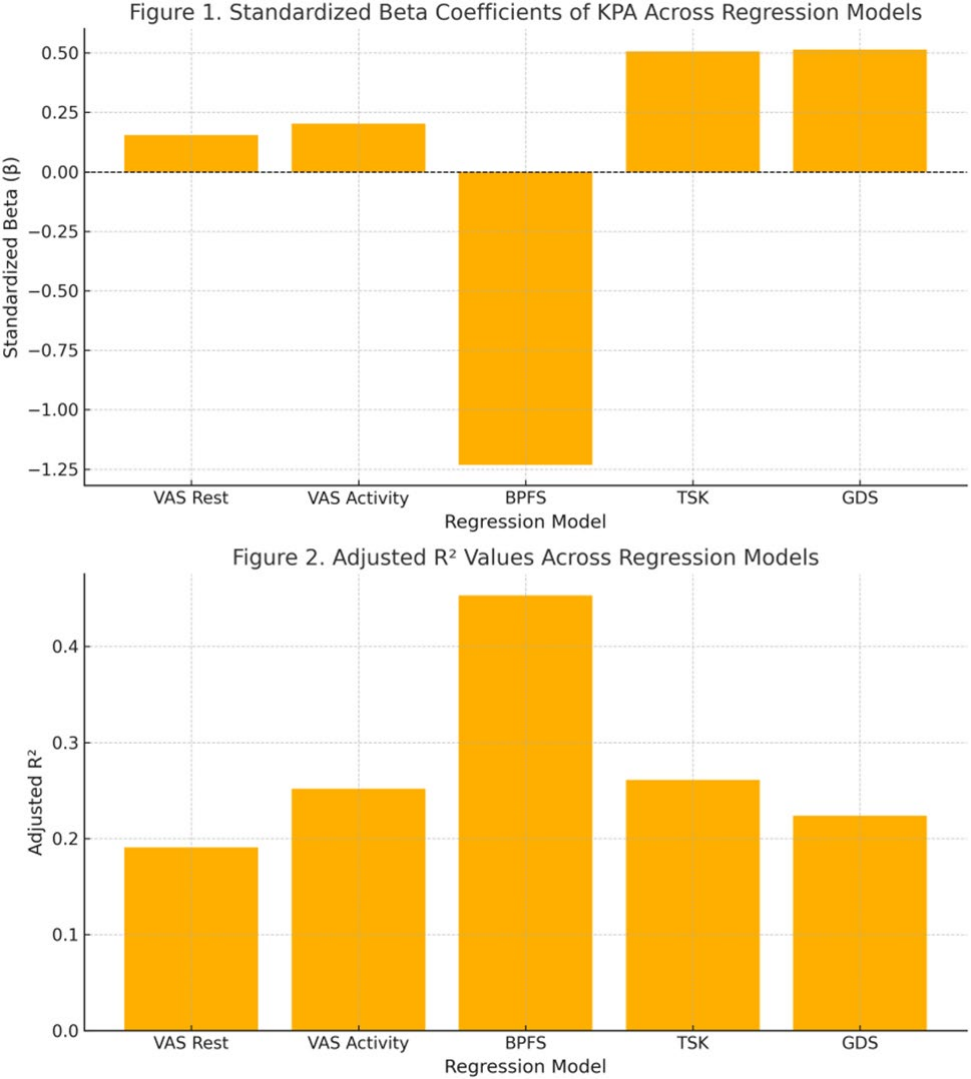
Predictive Role of KPA in Regression Models

Regression analysis was performed to explain the effect of social participation scores obtained from the KPA scale on other scales. A total of five regression models were established and all models were found to be statistically significant (*p* < 0.05). In addition, the relationship between the error terms in the models was examined and it was determined that there was no relationship between the error terms (no autocorrelation problem) (1.5 < DW < 2.5) (Fig. 1).

- According to Model 1, the change in KPA explained 19% of the change in patients’ VAS resting scores. A 1-unit increase in KPA caused a 0.15-unit increase in VAS resting scores.
- According to Model 2, the change in KPA explained 25% of the variance in patients’ VAS activity scores (R²=0.25). Additionally, a one-unit increase in KPA was associated with a 0.20-point increase in the VAS score.
- According to Model 3, change in KPA explains 45% of the change in BPFS score. A 1-unit increase in KPA causes a 1.23-unit decrease in functional low back pain score.
- According to Model 4, change in KPA explains 26% of the change in TSK scores. A 1-unit increase in KPA causes a 0.50-unit increase in TSK score.
- According to Model 5, KPA explains 22% of the change in GDS score. A 1-unit increase in KPA causes a 0.51-unit increase in GDS score.

In order to determine the strongest variables predicting KPA in older individuals with CNLBP, multiple linear regression analysis was performed using the stepwise method. BPFS is the strongest predictor of KPA (β =-0.676, *p* < 0.001). TSK (β = 0.276, *p* < 0.001), VAS activity (β = 0.172, *p* < 0.05) and GDS (β = 0.144, *p* < 0.05) were positively correlated with KPA (Table 4).

**Table 4:** Hierarchical multiple regression analysis predicting KPA scores

Model	Variables	β (Standardized)	t	*p*	Adj. *R*²	*R*² Change	F Change	Significance (*p*)
1	BPFS	-0.676	-9.761	< 0.001*	0.453	0.453	95.275	< 0.001
2	BPFS, TSK	-0.472 / 0.276	-6.781 / 3.941	< 0.001* / <0.001*	0.553	0.100	19.870	< 0.001
3	BPFS, TSK, VAS (Activity)	-0.385 / 0.234 / 0.172	-5.331 / 3.201 / 2.489	< 0.001* / 0.002* / 0.014*	0.585	0.032	6.228	0.014
4	BPFS, TSK, VAS (Activity), GDS	-0.330 / 0.216 / 0.155 / 0.144	-4.681 / 2.874 / 2.234 / 2.081	< 0.001* / 0.005* / 0.027* / 0.039*	0.603	0.018	4.305	0.039

*BPFS *Back Performance Functional Scale, *TSK *Tampa Scale of Kinesiophobia, *VAS (Activity) *Visual Analog Scale during activity, *GDS *Geriatric Depression Scale Statistical terms, *β *Standardized regression coefficient, *t *t-statistic, p = Significance level; Adj. R² = Adjusted R-squared; R² Change = Change in R-squared; F Change = F-statistic for model change; Significance (p) = p-value for model comparison *p < 0.05

## Discussion

This study evaluated the relationship between participation and physical and psychosocial factors in older adults with CNLBP. According to the evaluations, it was found that restricted participation of older individuals with CNLBP was associated with severe pain, increased depressive symptoms, increased kinesiophobia tendencies, and decreased functional capacity. These results are consistent with the view that physical and psychosocial factors in the subjective CNLBP experiences of older adults affect each other as discussed in the biopsychosocial model.

A study conducted in Egypt with patients with chronic low back pain reported a strong relationship between pain, depression, and functional disability [28]. Another study conducted in six low- and middle-income countries reported that as the severity of low back pain in older adults increased, the prevalence of major depressive disorder also increased [29]. A study conducted in Brazil with older individuals found a significant relationship between the severity of low back pain and the number of depressive symptoms; a link was also found between the number of disabilities and limitations caused by low back pain and the patients’ moderate depressive symptoms [30]. In a qualitative study conducted in the USA with older adults, it was found that the limitations experienced due to low back pain affected the patients physically (inability to perform routine tasks, sleep and exercise disorders), psychologically (sadness, irritability, fear of worsening health condition, hopelessness that the pain will go away or get better, social isolation) and individuals had difficulty in pursuing their hobbies [31]. In a qualitative study conducted in Australia with 11 male individuals with CNLBP, it was found that CLBP significantly limited their leisure time activities and that individuals experienced restrictions especially in their social areas [30]. In addition to international findings, recent studies conducted in Türkiye also support the relationship between pain, disability and functional performance in individuals with CNLBP. Uçar et al. (2021) reported a significant relationship between muscle volume, obesity, pain severity and disability scores in individuals with nonspecific low back pain [32]. Similarly, in a case-control study conducted by Özyurt et al. (2024), spinal mobility, aerobic capacity and functional mobility have been shown to be significantly reduced [33]. These national findings are consistent with the results of our study and confirm the multifaceted burden of CNLBP on both physical functioning and psychosocial well-being. In this study, a relationship was observed between pain and depression and functional disabilities/limitations in older adults with CNLBP. From this perspective, although they were conducted in countries with different levels of development, different communities and different regional pain areas, studies in the literature support a similar finding. Pain is an important factor that increases depression, functional disabilities and participation restrictions in patients. The main reason for this situation may be that pain is perceived by individuals as an unpleasant and disturbing situation in social, emotional and physical areas.

In a study conducted in Brazil among individuals aged 18–65, it was found that pain intensity was associated with kinesiophobia and functional disability level [34]. Another study conducted in Africa reported that disability scores due to low back pain increased with higher pain intensity and duration [35]. In a study conducted in Africa in 2021, it was reported that there was a relationship between kinesiophobia and low back pain severity, and that kinesiophobia increased as pain intensity increased [36]. Since a positive relationship was observed between low back pain and kinesiophobia in older adults, it was concluded that kinesiophobia may cause disability [35]. Similar findings were obtained in this study: kinesiophobia increased as pain intensity increased, which reduced the functional performance of the patients and increased their level of disability. When these results were evaluated within the framework of the Fear-Avoidance Model put forward by Vlaeyen and Linton (2000), they support the psychological cycle in which chronic pain leads to inactivity, depression and social isolation [37]. However, a study conducted in 2016 on older individuals with acute low back pain reported that there was no significant relationship between kinesiophobia and disability [36]. The reason why the results of this study differ from some studies in the literature may be cultural differences, the scales used are not similar to each other, and changes in pain duration.

In regression analyses, although the effect of social participation on functional status was quite strong (45.3%), the effect on pain (19–25%) and depression (22%) was more limited, which may be due to the differences between these variables. This is because participation is a concept directly related to functional status and overlaps with daily life activities and performance. On the other hand, since variables based on psychobiology such as depression or pain have multidimensional determinants, their explanation through participation alone is limited. This situation shows that participation has a more significant predictive effect, especially on physical functionality. In addition, when functional status, fear of movement, pain during activity and depression level were added to the model established in the hierarchical regression analysis, participation made an additional contribution to the prediction significantly, increasing the total explained variance to 60.3%. This may be due to the conceptual overlapping of the scales affecting the variance explanation rates. At the same time, it has been frequently emphasized in similar studies in the literature that chronic pain affects not only physiological but also psychological and behavioral components, and that this situation triggers movement avoidance behaviors [34, 35]. In light of these findings, it is understood that psychosocial components such as depression and kinesiophobia should also be addressed in CNLBP treatments, rather than focusing solely on pain reduction.

### Limitations of the study

There are some limitations related to this study. First, the cross-sectional and descriptive nature of the study may limit the establishment of a causal link in the study. Second, the fact that patients were collected from only one hospital and a specific region may limit the generalizability of the findings. The third limitation is that some psychosocial measurement tools used in the study, such as depression, kinesiophobia and social participation, touch on similar social-psychological themes despite assessing different constructs. Therefore, although the possibility of content overlap is low, there may be limited structural similarity between the measurements. Further studies should be supported by larger and more diverse samples and advanced statistical methods such as longitudinal designs and structural equation models.

## Conclusions

This study has shown that social participation in older adults with CNLBP has significant relationships not only with physical functioning but also with psychosocial conditions such as depression and kinesiophobia. This result provides an original contribution to the literature in that it shows that the level of social participation may be a key determinant in explaining the multidimensional effects of CNLBP. It is suggested that not only pain management but also strategies aimed at increasing social participation should be integrated in treatment and rehabilitation approaches, especially in older individuals. In addition, older individuals’ fear and avoidance attitudes towards treatment limit recovery and also lead to psychosocial burnout. Therefore, planning psychosocial interventions in the management of CNLBP is of critical importance.

In the context of the results, if some suggestions are put into action, the quality of life and social participation of older adults with CNLBP will increase. These are arrangements can be made for health professionals such as psychologists, physiotherapists and social workers to work together for the implementation of multidisciplinary interventions in health institutions. Managements in hospitals, care and rehabilitation centers should disseminate different programs to promote the functionality, mobility and social participation of older people. In addition, in the planning and implementation of these, artificial intelligence technology should be integrated into these programs recently to improve the health of older adults and the wider community. These innovative approaches can reduce both individual and community-based disability burden and cost.

## Supplementary Information

Supplementary Material 1.

Supplementary Material 2.

## Data Availability

It will be shared as an SPSS file by the author upon request.
