# Supplementary material for: Psychosocial and functional difficulties in older adults with chronic non-specific low back pain
Source: BMC Geriatr. 2026 Apr 13;26:766. doi: 10.1186/s12877-026-07420-y (PMC13220496; doi:10.1186/s12877-026-07420-y)
Supplement: Supplementary file 1 — Supplementary Material 1. [file 12877_2026_7420_MOESM1_ESM.pdf]

<u>**Keele Assessment of Participation**</u>

We are interested in some of the things that are necessary for you to live your life in the way you choose. We are particularly interested in how often these things are achieved in the way you would like.

When answering the questions, please think about the **past four weeks**. It does not matter if you require the help of other people or from gadgets and machines. We would simply like to know if the activity <u>**IS**</u> achieved to the extent that you want it to be.

Please read each statement below and put a cross in the box, which comes closest to how much you agree with the statement. Please put a cross in one box only for each line.

1. During the past 4 weeks, I have moved around in my home, <u>**as and when I have wanted.**</u>

All the time	Most of the time	Some of the time	A little of the time	None of the time
☐	☐	☐	☐	☐

2. During the past 4 weeks, I have moved around outside my home, <u>**as and when I have wanted.**</u>

All the time	Most of the time	Some of the time	A little of the time	None of the time
☐	☐	☐	☐	☐

3. During the past 4 weeks, my self-care needs (examples are washing, toileting, dressing, feeding, maintaining health) have been met, <u>**as and when I have wanted.**</u>

All the time	Most of the time	Some of the time	A little of the time	None of the time
☐	☐	☐	☐	☐

4. During the past 4 weeks, my home has been looked after, **<u>as and when I have wanted.</u>**

All the time	Most of the time	Some of the time	A little of the time	None of the time
☐	☐	☐	☐	☐

5. During the past 4 weeks, my things (belongings) have been looked after, **<u>as and when I have wanted.</u>**

All the time	Most of the time	Some of the time	A little of the time	None of the time
☐	☐	☐	☐	☐

6. Do you have any relatives, or other people, who depend on you?

Yes… ☐ No… ☐

If yes, during the past 4 weeks, were these people looked after, **<u>as and when you wanted?</u>**

All the time	Most of the time	Some of the time	A little of the time	None of the time
☐	☐	☐	☐	☐

7. During the past 4 weeks, I have met and spoken to other people **<u>as and when I have wanted.</u>**

All the time	Most of the time	Some of the time	A little of the time	None of the time
☐	☐	☐	☐	☐

8. During the past 4 weeks, I, or someone else on my behalf, have managed my money, **<u>as I have wanted.</u>**

All the time	Most of the time	Some of the time	A little of the time	None of the time
☐	☐	☐	☐	☐

9. Do you choose to take part in paid or voluntary work?

Yes…☐ No…☐

If yes, during the past 4 weeks, have you taken part in paid or voluntary work, **as and when you have wanted?**

All the time	Most of the time	Some of the time	A little of the time	None of the time
☐	☐	☐	☐	☐

10. Do you choose to take part in education or training courses?

Yes…☐ No…☐

If yes, during the past 4 weeks, have you taken part in education or training, **as and when you have wanted?**

All the time	Most of the time	Some of the time	A little of the time	None of the time
☐	☐	☐	☐	☐

11. Do you choose to take part in social activities?
(Examples of social activities are community and religious activities, meeting up with friends, going to clubs)

Yes…☐ No…☐

If yes, during the past 4 weeks, have you taken part in social activities, **as and when you have wanted?**

All the time	Most of the time	Some of the time	A little of the time	None of the time
☐	☐	☐	☐	☐
