# Supplementary material for: Psychosocial and functional difficulties in older adults with chronic non-specific low back pain
Source: BMC Geriatr. 2026 Apr 13;26:766. doi: 10.1186/s12877-026-07420-y (PMC13220496; doi:10.1186/s12877-026-07420-y)
Supplement: Supplementary file 2 — Supplementary Material 2. [file 12877_2026_7420_MOESM2_ESM.pdf]

KEELE KATILIM DEĞERLENDİRMESİ (KKD)

Hayatınızı seçtiğiniz şekilde yaşamanız için gerekli olan bazı işlerle ilgileniyoruz. Özellikle bu işlerin, istediğiniz şekilde ne sıklıkla gerçekleştiği ile ilgileniyoruz.

Soruları cevaplarken lütfen son dört haftayı düşünün. Başkalarının yardımına, araç gereçlere ve makinelere ihtiyacınız olup olmadığı önemli değildir. Biz sadece aktivitenin olmasını istediğiniz ölçüde gerçekleşip gerçekleşmediğini bilmek istiyoruz.

Lütfen aşağıdaki her ifadeyi okuyun ve ifadeye ne kadar katıldığınıza ait kutuya çarpı işareti koyun. Lütfen her satırda sadece bir kutuya çarpı koyun.

1. Son 4 hafta boyunca, evimin içinde istediğim zaman ve istediğim gibi dolaştım.

Her zaman	Çoğu zaman	Bazen	Nadiren	Hiçbir zaman
☐	☐	☐	☐	☐

2. Son 4 hafta boyunca, evimin dışında istediğim zaman ve istediğim gibi dolaştım.

Her zaman	Çoğu zaman	Bazen	Nadiren	Hiçbir zaman
☐	☐	☐	☐	☐

3. Son 4 hafta boyunca, öz bakım ihtiyaçlarımı (örneğin; yıkanma, tuvalet, giyinme, beslenme, sağlığın korunması) istediğim zaman ve istediğim gibi karşıladım.

Her zaman	Çoğu zaman	Bazen	Nadiren	Hiçbir zaman
☐	☐	☐	☐	☐

4. Son 4 hafta boyunca, evimle istediğim zaman ve istediğim gibi ilgilendim.

Her zaman	Çoğu zaman	Bazen	Nadiren	Hiçbir zaman
☐	☐	☐	☐	☐

5. Son 4 hafta boyunca, kişisel eşyalarımla istediğim zaman ve istediğim gibi ilgilendim.

Her zaman	Çoğu zaman	Bazen	Nadiren	Hiçbir zaman
☐	☐	☐	☐	☐

6-Size bağımlı olan akrabalarınız veya başka insanlar var mı?

Evet ☐ Hayır ☐

Evet ise, son 4 hafta boyunca, bu insanlarla istediğiniz zaman ve istediğiniz gibi ilgilendiniz mi?

Her zaman ☐ Çoğu zaman ☐ Bazen ☐ Nadiren ☐ Hiçbir zaman ☐

7. Son 4 hafta boyunca, başka insanlarla istediğim zaman ve istediğim gibi buluştum ve konuştum.

Her zaman ☐ Çoğu zaman ☐ Bazen ☐ Nadiren ☐ Hiçbir zaman ☐

8. Son 4 hafta boyunca, parasal işlerimi (banka, fatura, maaş,…) istediğim zaman ve istediğim gibi halledebildim.

Her zaman ☐ Çoğu zaman ☐ Bazen ☐ Nadiren ☐ Hiçbir zaman ☐

9. Ücretli veya gönüllü olarak bir işte yer almayı ister misiniz?

Evet ☐ Hayır ☐

Evet ise, son 4 hafta boyunca, ücretli veya gönüllü bir işte istediğiniz zaman ve istediğiniz gibi yer aldınız mı?

Her zaman ☐ Çoğu zaman ☐ Bazen ☐ Nadiren ☐ Hiçbir zaman ☐

10. Eğitim veya öğretim kurslarında yer almayı ister misiniz?

Evet ☐ Hayır ☐

Evet ise, son 4 hafta boyunca, eğitim veya öğretimde istediğiniz zaman ve istediğiniz gibi yer aldınız mı?

Her zaman	Çoğu zaman	Bazen	Nadiren	Hiçbir zaman
☐	☐	☐	☐	☐

11. Sosyal aktivitelerde yer almayı ister misiniz? (Sosyal aktivite örnekleri; topluluk ve dini etkinlikler, arkadaşlar ile buluşma, derneklere gitmektir.)

Evet ☐ Hayır ☐

Evet ise, son 4 hafta boyunca, sosyal aktivitelerde istediğiniz zaman ve istediğiniz gibi yer aldınız mı?

Her zaman	Çoğu zaman	Bazen	Nadiren	Hiçbir zaman
☐	☐	☐	☐	☐
